# Experience Applying the Guidelines for Reporting Reliability and Agreement Studies (GRRAS) Indicated Five Questions Should Be Addressed in the Planning Phase from a Statistical Point of View

**DOI:** 10.3390/diagnostics8040069

**Published:** 2018-09-24

**Authors:** Oke Gerke, Sören Möller, Birgit Debrabant, Ulrich Halekoh, Odense Agreement Working Group

**Affiliations:** 1 Department of Nuclear Medicine, Odense University Hospital, J.B. Winsløws Vej 4, 5000 Odense C, Denmark; 2 Department of Clinical Research, University of Southern Denmark, Winsløwparken 19, 3. sal, 5000 Odense C, Denmark; moeller@health.sdu.dk; 3 Odense Patient data Explorative Network, Odense University Hospital, J.B. Winsløws Vej 9a, 5000 Odense C, Denmark; 4 Department of Public Health, Epidemiology and Biostatistics, University of Southern Denmark, J.B. Winsløws Vej 9b, 5000 Odense C, Denmark; bdebrabant@health.sdu.dk (B.D.); uhalekoh@health.sdu.dk (U.H.)

**Keywords:** agreement, guideline, interrater, intrarater, reliability, reproducibility

## Abstract

The Guidelines for Reporting Reliability and Agreement Studies (GRRAS) were proposed in 2011 to support transparent and accurate reporting. These studies may be conducted with the primary aim of estimating reliability and/or agreement itself, but are more often than not part of larger diagnostic accuracy studies, clinical trials, or epidemiological studies. As such, the study design may be compromised in terms of practicability issues, preventing the collection of sufficient results. We presented an example from a consultancy with a difficult mission and discussed five questions that concern the very nature of such a study (agreement vs. reliability; intra- vs. interrater), the rater population, explanatory factors in a multivariable model, and the statistical analysis strategy. Discussion of such basic methodological and statistical questions must take place before an investigation is started in order to ensure adequate data collection, to predict possible complications in the study, to plan sufficient statistical analyses, and to request timely assistance from an experienced statistician. GRRAS and its accompanying checklist of 15 items proved to be most helpful. Hopefully, our commentary will help improve the planning of agreement and reliability studies, which, in turn, will then be more focused, more appropriate, and more easily reported using GRRAS.

## 1. Introduction

In 2011, the Guidelines for Reporting Reliability and Agreement Studies (GRRAS) were published, as studies of interater/intrarater reliability and agreement were often found to be incomplete and inadequate, and widely accepted criteria, standards, or guidelines for reporting of such studies were lacking in health care and medical science [1]. Today, GRRAS is one of many guidelines supporting transparent and accurate reporting and has become part of the Enhancing the Quality and Transparency of Health Research (EQUATOR) network [2]. Having worked with GRRAS over the recent years in statistical consultancy for PhD students and clinical researchers in the health sciences, we found the 15 points to be addressed most helpful. However, we recommend five questions that should be discussed in the planning phase from a statistical point of view in order to secure an agreement and/or a reliable study that successfully illuminates what it intends to, and to make the researcher aware of the prospects and limitations of such a study from the start. These questions reinforce or relate to specific items from GRRAS and substantiate the guidelines’ claim that “*Researchers should clearly state a priori their assumptions, why a certain approach was chosen, and what was intended to be demonstrated.*”

In the following, an example from our consultancy is presented in which interrater agreement was sought to be investigated at the end of the study, but this was impossible due to a study design that was compromised on practicability issues. We discuss the five questions that supplement the related GRRAS items and return briefly to the introductory example in light of the five questions. Finally, a table sets out a set of characteristics of any agreement and/or reliability study that can be used both in the planning phase and in consultancy.

## 2. An Example of a Consultancy with a Difficult Mission

Intravascular optical coherence tomography (IVOCT) is an imaging technique that is used to analyze the underlying cause of cardiovascular disease (e.g., [3]). A local study in Odense, Denmark, concerned the validation of IVOCT with histology of the atherosclerosis of the coronary arteries. Five vessels from three patients were analyzed, and 175 cross-sections were considered in every vessel. At every cross-section, three stains were placed and arterial permeability was measured. Raters A and B evaluated 3 stains × 175 cross-sections × 5 vessels = 2625 stains in histological examinations. Rater C correspondingly assessed three images per cross-section with IVOCT.

After completing the data collection and various exploratory analyses, the student sought help in our consultancy. The student had discovered that the Bland–Altman limits of agreement were not applicable due to the correlation structure of the data, and the data suggested substantial systematic differences between rater C employing IVOCT on the one hand and raters A and B on the other hand. The latter raised the question whether the comparison of raters A and B vs. C was meaningful. Finally, the agreement analyses of the data were, to the best of our knowledge, not reported.

## 3. Do You Want to Investigate Interrater/Intrarater Agreement or Reliability? (Item 1: Identify in Title or Abstract That Interrater/Intrarater Agreement or Reliability Was Investigated)

Studies of agreement and/or reliability are, in our experience, more often than not part of larger diagnostic accuracy studies, clinical trials, or epidemiological studies in which agreement and/or reliability are reported as a quality control, using data from the main study. Unfortunately, the planning of such sub-studies regularly fails to precede the data collection process, and researchers are not aware of the complementary nature of intrarater variability analysis on the one hand (focusing on one specific observer—a question of internal validity) and interrater variability analysis on the other hand (investigating differences between several observers—a question of external validity). Central terms are regularly used interchangeably, and the conceptual difference of agreement (targeting the degree to which scores or ratings are identical) and reliability (relating to the ability of the scores of a measuring device to differentiate among subjects or objects) is often unclear, but has direct implications on which statistical measures to use (Table 1, with data from [4,5,6,7]; see also Table 2 in [1]). 

Accurate and reliable measurements serve as the basis for evaluation in the social, behavioral, physical, biological, and medical sciences [8]. As agreement analysis implies the estimation of the measurement error in repeated measurements, whereas reliability assessment reflects the distinguishability of study objects despite measurement errors, a reliability parameter (such as, for instance, an intraclass correlation coefficient) approaches 1 if the measurement error is small compared to the variability between study objects. In contrast, if the measurement error is large compared to the variability between study objects, the reliability parameter will be smaller, as the discrimination is affected by the measurement error [9]. The larger the variability between study objects, the larger the reliability parameter will be due to the decreasing influence of the measurement error itself, and vice versa [10]. To this end, agreement and reliability measures can be considered as absolute and relative measures, addressing different questions [9].

## 4. Who Represents the Rater Population of Interest? (Item 4: Specify the Rater Population of Interest (If Applicable))

Discussions on intra- and interrater variability studies often start as follows: “How many raters should I employ?”—“Well, it depends on what you want to show.” The underlying question to be answered is whether the researcher seeks a variability assessment for one or more specific raters—as these are the only ones rating in daily routine—or whether the included raters represent a wider rater population of interest. The latter case applies, for instance, in multicenter studies or when the results will be generalized to other studies. The choice to be made here affects whether to treat the variable ‘rater’ in a generalized linear model as a fixed or random effect, respectively. Consequently, differences between raters can either be estimated and the respective uncertainty quantified by means of 95% confidence intervals, or ‘rater’ becomes a variance component of the model. Either way, appropriate repeatability coefficients can be derived based on the model. These values quantify within which limits, on average, 95% of subjects will fall when readings are done under the same conditions [11]. 

## 5. Which Factors Will Your Model Account for? (Item 6: Explain How the Sample Size Was Chosen. State the Determined Number of Raters, Subjects/Objects, and Replicate Observations)

Statistical modelling issues and especially a priori sample size calculations are more challenging than GRRAS suggests. The sample size chosen (if motivated at all) depends on both the number and scale of predictor variables (continuous, categorical, or binary) in the model. In many situations, and as a rule-of-thumb, around 10 to 20 observations per predictor variable are sufficient to accurately estimate the coefficients of a regression model [12,13]. The number of raters and the decision to consider ‘rater’ as a fixed or random factor influences the sample size directly, since sufficiently accurately estimating variance components requires more observations compared to fixed effects. 

Adding interaction terms to the model in order to account for, for instance, observer × subject interaction (since the difference between observers may vary from subject to subject) increases the number of coefficients to be estimated further [11,14]. To this end, repeated measurements (also called replicates) on every subject by every observer are necessary. The most parsimonious study will employ two observers with two measurements on every subject. Alternatively, subjects can be randomized to having one observer performing two replicated measurements with the other observer performing the assessment just once; thereby, both intra-observer variability for the two observers and inter-observer variability can be evaluated. In contrast, Carstensen’s broad recommendation is 50 subjects with three replicates on each method in a method comparison study [14].

It must be noted that the abovementioned rule-of-thumb of around 10 to 20 observations per predictor variable (or events in connection with the regression analysis of time-to-event or binary endpoints) is controversial and has its advocates [15,16,17] and opponents [18,19]. Sitch and colleagues produced a shiny app which allows the precision of estimates to be estimated for given numbers of raters, observations, and replications [20].

## 6. Which Indices for Reliability or Agreement Are You Aiming for? What Is Your Statistical Analysis Plan? (Item 13: Report Estimates of Reliability and Agreement Including Measures of Statistical Uncertainty and Item 10: Describe the Statistical Analysis)

In prolongation of the first question above, the aims of an agreement and/or reliability study need to be translated to and addressed with appropriate measures (see Table 2 in [1]). The two preceding questions regarding raters and factors underline the necessity of the a priori planning of the statistical analysis strategy in order to collect data accordingly and appropriately (e.g., number of raters, replications, other factors). This allows the building of a model from which all relevant measures can be derived. As usual, this requires the aims, design, data collection, and analysis to follow each other chronologically (e.g., [21]). When reversing this order, for example, by collecting the data first and determining the analysis strategy later, the analysis runs the risk of being data-driven and it may not be possible for the model to be formulated and fitted as would be desirable for its intended purpose.

## 7. Revisiting the Introductory Example

The considered agreement analysis targeted the measurement error of arterial permeability itself, whereas reliability analysis would have concerned the distinguishability of the vessel stains despite the abovementioned measurement error. Moreover, a discussion of our five questions at the onset of the study would most likely have led to a discussion of intrarater analysis for rater C, as well as of rater A or B, since two raters with repeated measurements would have been beneficial over three raters with non-replicated measurements when assessing variability. However, rater C was an expert reader who was available only for single, i.e., non-repeated readings. Moreover, a hierarchical statistical model with stains nested in the cross-sectional position, nested in the vessel, and nested in the patient could have been considered, implying a modified data collection plan in order to make the imbalances less extreme (fewer cross-sectional positions, but more vessels, and more patients). However, retaining many stains from few vessels was more practical and less expensive than aiming for fewer stains from a larger number of vessels.

## 8. Final Remarks

Admittedly, our primary focus is the assessment of intra- and interrater variability of continuous measurements where appropriate measures such as repeatability coefficients and intraclass correlation coefficients are preferably derived from one joint model. Generalized linear models may likewise be formulated for ordinal or binary outcomes, as multilevel mixed-effects ordered logistic models or by employing the logit link function, respectively. However, especially the interpretability of repeatability coefficients is then less clear. 

GRRAS has proven to be most helpful, but consulting on agreement and reliability studies resembles a continuous awareness campaign. We hope that our reflections on GRRAS from a statistical point of view will help improve the planning of such studies which, in turn, will then be more focused, more appropriate, and more easily reported using GRRAS.

## Figures and Tables

**Table 1:** Main characteristics template for an agreement and/or reliability study.

Characteristic	Premature Atrial Complexes and Atrial Fibrillation in Ischemic Stroke Patients [4]	[15O]Water Myocardial Flow Reserve PET and CT Angiography by Hybrid Full 3D PET/CT [5]	Bone Mineral Density Measurements around Acetabular Cup [6]	Dual Time PET/CT in the Preoperative Assessment of Ovarian Cancer [7]
Agreement vs. reliability	Agreement	Agreement & reliability	Agreement & reliability	Agreement
Method(s)	Echocardiography	[15O]water PET	Single and dual-energy CT	[^18^F]DG PET/CT
Outcome	LASI (cont.)	Coronary flow reserve (stress; rest; myocardial flow reserve—cont.)	Bone mineral density (cont.)	SUVmax (cont.)
Rater(s)	2 physicians	2 physicians	2 radiographers	1 physician
No. of measurements (replicates)	2 by each physician	2 by each physician	2 by each radiographer	2
Intra- or inter-rater analysis	Intra & inter	Intra & inter	Intra & inter	Intra
Item (i.e., observational unit)	24 patients with one image each	44 patients with 44 global measurements and 3 × 44 segmental measurements in 3 arteries (LAD, LCX, RCA)	24 acetabular cups inserted in porcine hip specimens	30 patients with one image each
Other factors (fixed; random)	None	None	None	None
Analysis	BA LoA, RC	BA LoA, ICC, RC	BA LoA, ICC, RC	BA LoA, RC

BA LoA: Bland-Altman limits of agreement, cont.: continuous, CT: computed tomography, [18F]DG: 2-deoxy-2-[18F]fluoro-D-glucose, ICC: intraclass correlation coefficient, LAD: left anterior descending artery, LASI: left atrial sphericity index, LCX: left circumflex coronary artery, [15O]: Oxygen-15 water, PET: positron emission tomography, RC: repeatability coefficient, RCA: right coronary artery, SUVmax: maximum standardized uptake value.
